# Postural instability revealing infective endocarditis secondary to severe mitral stenosis: A case report with literature review

**DOI:** 10.1016/j.amsu.2021.103131

**Published:** 2021-12-02

**Authors:** Raid Faraj, Zaineb Bourouhou, Houda Belhoussine, Asmae Bouamoud, Hasna Rami, Mohamed Cherti

**Affiliations:** Department of Cardiology B, CHU Ibn Sina, Rabat, Morocco

**Keywords:** Mitral stenosis, Infective endocarditis, Vegetation, Embolic stroke

## Abstract

**Introduction and importance:**

Infective endocarditis is a severe infection of the endocardial surface of the heart. One or more heart valves can be infected. However infective endocarditis complicating mitral stenosis is rare. It can be revealed by several and various symptoms such as fever and cardiac murmurs but also by complications such as focal neurological complaints

**Case presentation:**

We report a case of a febrile postural instability as the primary presentation of an infective endocarditis secondary to a severe mitral stenosis in a young patient with a history of mitral stenosis for which he benefited from percutaneous mitral dilation. The diagnosis was based on the modified Duke criteria. In this case, the treatment was based mainly on antibiotic therapy. The outcome was favorable; with a clinical, biological and radiological improvement. The patient was subsequently referred to the cardiovascular department for surgical treatment of his valve disease.

**Clinical discussion:**

Rheumatic heart disease is the main cause of mitral stenosis and its prevalence is higher in developing nations than in the rest of the world, yet only few articles have reported infective endocarditis as a complication of mitral stenosis.

**Conclusion:**

Mitral stenosis is rarely complicated by infective endocarditis, but this diagnosis should not be excluded in developing countries, particularly because of its high prevalence. To that end, clinicians should recognize its symptoms and complications and act accordingly to allow early treatment.

## Highlights

• Mitral stenosis is rarely complicated by infective endocarditis.
• Recognizing infective endocarditis at an early stage is essential.
• Antimicrobial therapy is the backbone therapy of infective endocarditis along with surgery management, in some cases of complicated IE.

## 1 Introduction

Up to 10 million children and young adults have mitral stenosis worldwide [1]. Its prevalence in developed countries is low, in contrast with developing countries [2]. It's considered as the most commonly acquired heart disease in individuals under the age of 25 [2]. Despite the efforts of the medical community to eradicate it in many countries of the globe, over 288 348 deaths are deplored each year [2].

The clinical presentation of mitral stenosis is related to its severity. Many patients may remain asymptomatic [3]. Infective endocarditis as a complication of mitral stenosis is less recognized and described. This report aims to present the case of an infective endocarditis complicating severe mitral stenosis and revealed by febrile postural instability. Recognizing infective endocarditis at an early stage is essential, allowing the initiation of treatment and avoiding a broad array of systemic complications.

To the best of our knowledge, this is the first published case of blood culture-negative infective endocarditis (BCNIE) secondary to a severe mitral stenosis and revealed by a febrile postural instability.

Our case report was written according to the CARE guidelines [4].

## 2 Case presentation

A 45-year-old male was admitted to the emergency department with postural instability and dysarthria. To lessen his instability and avoid to fall, the patient widened his support polygon. He had also reported dyspnea at effort, which occurred 3 days prior to his admission. He had a history of rheumatic mitral stenosis, since 2005, for which he benefited from a percutaneous mitral dilation in the same year. He also reported a Penicillin allergy.

Initial examination found the patient conscious. His heart rate was 125 b/m, blood pressure was 135/85 mm Hg. He was polypneic and orthopneic with a respiratory rate of 28 breaths/min, an O2 saturation of 96% on ambient air with the presence of bilateral crackles. He had a fever measured at 39.5C. Cardiac

auscultation revealed a low-pitched diastolic rumble, well heard at the apex. The neurologic examination revealed unsteady gait and the patient was unable to perform Romberg's test.

**Fig. 1:**
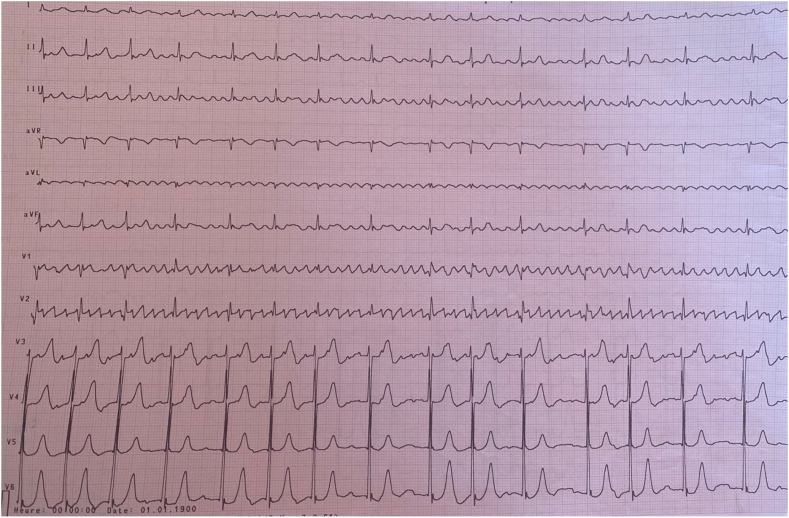
ECG showing coarse-mesh atrial fibrillation.

**Fig. 2:**
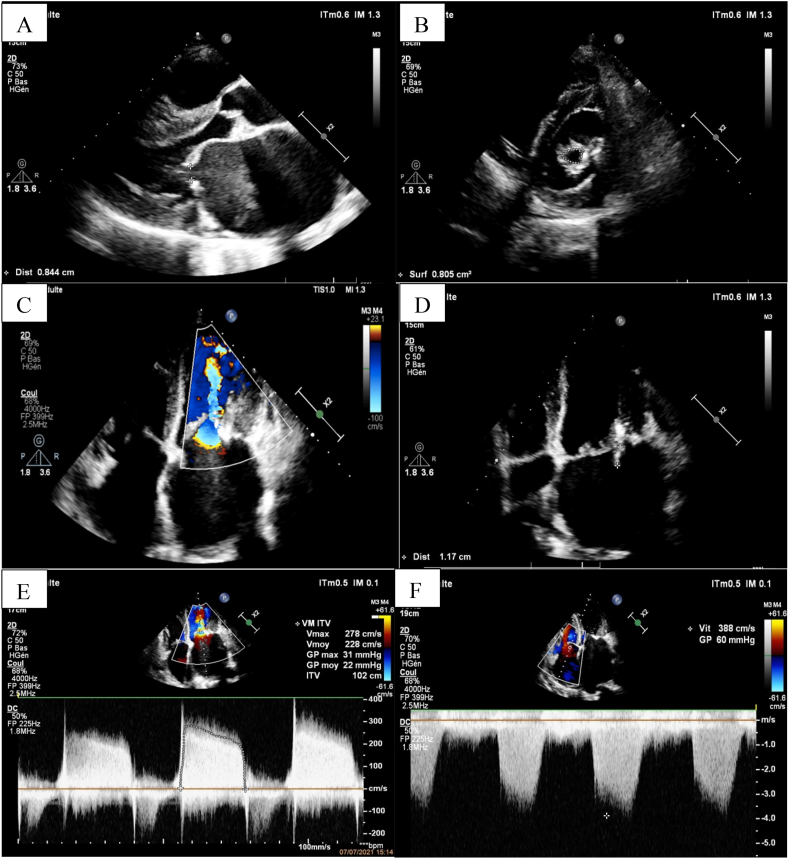
TEE findings: (A) parasternal long axis view showing rheumatic changes of the mitral valve with a “dog leg deformity” of the anterior mitral leaflet and an important enlargement of the left atrium. (B) parasternal short axis view showing an important mitral valve area stenosis, evaluated at 0,8cm2. (C) Apical long axis view with color flow Doppler showing a high velocity narrow "jet" across a stenotic mitral valve during diastole. (D) Apical long axis view showing a mobile vegetation measuring 11,7mm of length located in the posterior leaflet of the mitral valve. (E) Continuous wave Doppler with a mean gradient of 22 mm Hg. (F) Continuous wave Doppler with an important PASP. (For interpretation of the references to color in this figure legend, the reader is referred to the Web version of this article.)

The ECG showed coarse-mesh atrial fibrillation with an average ventricular rate of 90 cycles per minute (Fig. 1). No abnormalities were detected on the chest x-ray. Transthoracic echocardiogram (TTE) found rheumatic changes of the mitral valve including: commissural fusion and thickening, producing “dog leg deformity” of the anterior mitral leaflet (Fig. 2-A). The mitral valve area was 0,8 cm2 (Fig. 2-B) and the pressure gradient across the mitral valve was 22 mmhg (Fig. 2-E). We noted a mobile vegetation measuring 11,7 mm of length, located in the posterior leaflet of the mitral valve (Fig. 2-C). The left atrium was dilated at 47 cm2 while size and function of the left ventricle was normal. Pulmonary arterial systolic pressure (PASP) was important (Fig. 2-F) and the filling pressures of the left ventricle were elevated. In addition to that, we reported moderate aortic stenosis and regurgitation and mitral regurgitation at grade A.

**Fig. 3:**
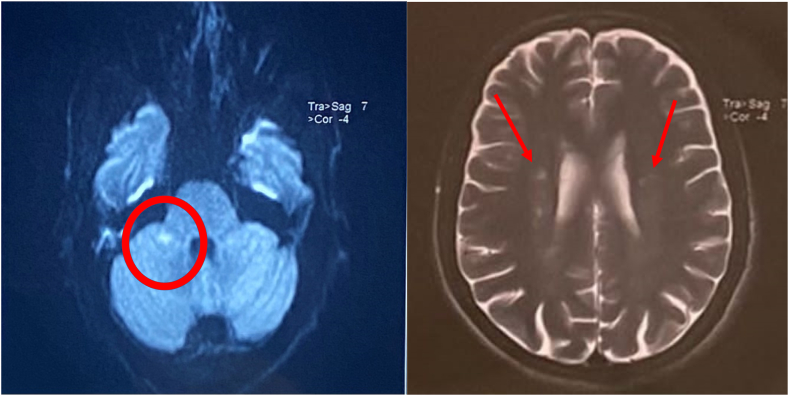
Axial T2-weighted magnetic resonance imaging showing a recent ischemic stroke involving the right peduncular territory (A) associated with chronic lacunar infarcts (B).

Three sets of blood cultures were made, coming back negative as well as the HACEK organism's serologies (Haemophilus; Actinobacillus; Cardiobacterium; Eikenella; and Kingella). It was decided to perform a brain MRI based on the clinical findings. It showed a recent ischemic stroke involving the right peduncular territory associated with chronic lacunar infarcts (Fig. 3).

Signs of inflammatory response were noted, high white blood cell count at 14 770/mm3 in addition to C reactive protein (CRP) at 67 mg/l, Ferritin was elevated at 839 μg/l. The kidney function was normal. Further investigations were realized to precise the extension of infective endocarditis, all coming back normal. No point of entry for the pathogen was detected.

The patient was admitted to the cardiology unit. The diagnosis of infective endocarditis was made, based on the modified Duke criteria (1 major clinical criteria +3 minors clinical criteria). Given the negative blood culture endocarditis and the history of penicillin allergy, it was decided to start an antibiotic therapy combining: Vancomycin at 30mg/kg/day for 6 weeks (Vancomycin serum concentrations of 15–20 mg/L were aimed) and Gentamicin at 3mg/kg/day for 2 weeks, in addition to intravenous diuretics (80 mg of Furosemide as bolus followed by a maintenance dose of 40mg/12hours) with a strict control of kalaemia. Finally, for his supraventricular arrythmia, Enoxaparin was started at a curative dose (100 UI/kg/12h). The tolerability was good and no adverse events were reported.

Within 4 days, fever disappeared. The postural instability and the dysarthria were less important than in his admission. Routine TTEs showed a regression of the vegetation and a normalization of the left ventricular pressures. The patient was satisfied after the improvement of his clinical condition. He was addressed after that to the cardiovascular surgery department for a surgical treatment of his valve disease.

## 3 Discussion

Acquired valvular disorders are considered as an important risk factor of infective endocarditis. Indeed, a study based on a danish registry between 2005 and 2015 confirmed that patients with a valve disorder had a higher risk of developing infective endocarditis than matched controls [5].

In contrast to mitral regurgitation which exposes to a five to eight times higher risk of developing infective endocarditis, mitral stenosis is rarely incriminated [6,7].

Few data established a relation between infective endocarditis and mitral stenosis. In a recent case published in the journal of the American college of cardiology, a 28-year-old woman, with no history of rheumatic disease, presented functional mitral stenosis as a result of severe bacterial endocarditis, complicated with bilateral ischemic toes [8]. However, cases of infective endocarditis complicating mitral stenosis are very rare. In an article published in the Japanese heart journal, authors have described a case of mitral stenosis complicated with seronegative Brucella endocarditis in a 30 years old woman complaining of palpitations, dyspnea, fever, and fatigue, unlike our case, there was no history of rheumatic disease and the clinical presentation was not dominated by neurological symptoms.

As it is well known, mitral stenosis is a valvular disorder, that causes obstruction to blood flow from the left atrium to the left ventricle. While the left ventricle remains unaffected, there are increased pressures in the left atrium, pulmonary vasculature, and the right heart cavities [3]. Unlike other valvular disorders, It's mainly caused by rheumatic heart disease [9].

Its complications are multiple, including atrial fibrillation, thromboembolism, heart failure and rarely infective endocarditis. In a cohort including 3343 children and adults with rheumatic mitral stenosis, the frequency of infective endocarditis ranged from 2,3 to 5,7% [10].

The diagnosis of mitral stenosis is suspected in a patient with a history of rheumatic heart disease and suggestive symptoms of mitral stenosis. The presence of atrial fibrillation is the most important finding on electrocardiogram in patients with mitral stenosis. Chest x-ray may be normal in non-advanced cases especially in younger patients. However, in older patients with severe mitral stenosis, marked enlargement of the left atrium is noted. Transthoracic echocardiography (TTE) is used to confirm the diagnosis and evaluate the hemodynamic severity. When infective endocarditis is suspected, we apply the modified Duke criteria.

The vegetation size is a major risk factor of embolization [11]. It was confirmed in a meta-analysis published in 2018, that found that patients with IE and vegetation size >10 mm had a high risk of embolic events and in-hospital mortality [12].

Embolization may even occur before the diagnosis of IE, as its reported in our case. In fact, neurological symptoms were in the foreground, secondary to cerebellar embolization [13].

Antimicrobial therapy is the backbone therapy of infective endocarditis along with surgery management, in some cases of complicated IE [14].

In patients with culture-negative native valve endocarditis (NVE), antimicrobial therapy should cover both gram-negative and gram-positive organisms. Indeed, the American heart association recommends the use of Vancomycin and Cefepime as initial regimen [15], while the European society of cardiology recommends the use of Ampicillin with Oxacillin and Gentamycin [16]. Both agree that patients with blood culture-negative infective endocarditis should be treated in consultation with an infectious disease specialist. We chose, however, to treat our patient using Vancomycin with Gentamycin, due to his penicillin allergy history.

After 6 weeks of treatment and after a multidisciplinary discussion, the patient was addressed to the cardiovascular surgery department for a surgical treatment of his valve disease. In fact, early surgery may be beneficial to reduce the risk of embolism in patients with large vegetations (>10 mm) [15,16].

## 4 Conclusion

Our case highlights an uncommon presentation of blood culture-negative infective endocarditis (BCNIE) secondary to severe mitral stenosis. Early diagnosis and treatment are crucial to avoid a broad array of systemic complications.

## Ethical approval

N/a.

## Sources of funding

None.

## Author contribution

Raid Faraj: Study concept, Data collection, Data analysis, Writing the paper.Zaineb Bourouhou: Contributor, Data collection Houda Belhoussine: Contributor, Data collection Asmae Bouamoud: Contributor Hasna Rami: Contributor Mohamed Cherti: Supervision and data validation.

## Consent

Written informed consent was obtained from the patient for publication of this case report and accompanying images. A copy of the written consent is available for review by the Editor-in-Chief of this journal on request.

## Research registration

N/a.

## Guarantor

Raïd Faraj.

## Provenance and peer review

Not commissioned, externally peer-reviewed.

## Declaration of competing interest

None.
