# Supplementary material for: Postural instability revealing infective endocarditis secondary to severe mitral stenosis: A case report with literature review
Source: Ann Med Surg (Lond). 2021 Dec 2;72:103131. doi: 10.1016/j.amsu.2021.103131 (PMC8649078; doi:10.1016/j.amsu.2021.103131)
Supplement: Multimedia component 1 [file mmc1.pdf]

CARE
case report guidelines

CARE Checklist of information to include when writing a case report

(cc) BY-NC-ND

Topic	Item	Checklist item description	Reported on Line
Title	1	The diagnosis or intervention of primary focus followed by the words “case report”	2-3
Key Words	2	2 to 5 key words that identify diagnoses or interventions in this case report, including "case report"	31
Abstract (no references)	3a	Introduction: What is unique about this case and what does it add to the scientific literature?	9-13
	3b	Main symptoms and/or important clinical findings	14-17
	3c	The main diagnoses, therapeutic interventions, and outcomes	18-21
	3d	Conclusion—What is the main “take-away” lesson(s) from this case?	26-30
Introduction	4	One or two paragraphs summarizing why this case is unique (**may include references**)	34-48
Patient Information	5a	De-identified patient specific information.	51
	5b	Primary concerns and symptoms of the patient.	52-54
	5c	Medical, family, and psycho-social history including relevant genetic information	54-56
	5d	Relevant past interventions with outcomes	54-56
Clinical Findings	6	Describe significant physical examination (PE) and important clinical findings.	57-63
Timeline	7	Historical and current information from this episode of care organized as a timeline	
Diagnostic Assessment	8a	Diagnostic testing (such as PE, laboratory testing, imaging, surveys).	64-85
	8b	Diagnostic challenges (such as access to testing, financial, or cultural)	
	8c	Diagnosis (including other diagnoses considered)	
	8d	Prognosis (such as staging in oncology) where applicable	not applicable
Therapeutic Intervention	9a	Types of therapeutic intervention (such as pharmacologic, surgical, preventive, self-care)	88-94
	9b	Administration of therapeutic intervention (such as dosage, strength, duration)	88-94
	9c	Changes in therapeutic intervention (with rationale)	
Follow-up and Outcomes	10a	Clinician and patient-assessed outcomes (if available)	96-101
	10b	Important follow-up diagnostic and other test results	96-101
	10c	Intervention adherence and tolerability (How was this assessed?)	94-95
	10d	Adverse and unanticipated events	94-95
Discussion	11a	A scientific discussion of the strengths AND limitations associated with this case report	104-109
	11b	Discussion of the relevant medical literature **with references**.	104-119
	11c	The scientific rationale for any conclusions (including assessment of possible causes)	
	11d	The primary “take-away” lessons of this case report (without references) in a one paragraph conclusion	159-162
Patient Perspective	12	The patient should share their perspective in one to two paragraphs on the treatment(s) they received	98-99
Informed Consent	13	Did the patient give informed consent? Please provide if requested	Yes ☑ No ☐
